# Loss of ATP-Sensitive Potassium Channel Surface Expression in Heart Failure Underlies Dysregulation of Action Potential Duration and Myocardial Vulnerability to Injury

**DOI:** 10.1371/journal.pone.0151337

**Published:** 2016-03-10

**Authors:** Zhan Gao, Ana Sierra, Zhiyong Zhu, Siva Rama Krishna Koganti, Ekaterina Subbotina, Ankit Maheshwari, Mark E. Anderson, Leonid V. Zingman, Denice M. Hodgson-Zingman

**Affiliations:** 1 Department of Internal Medicine, Carver College of Medicine, University of Iowa, Iowa City, Iowa, United States of America; 2 François Abboud Cardiovascular Research Center, University of Iowa, Iowa City, Iowa, United States of America; 3 Fraternal Order of Eagles Diabetes Research Center, University of Iowa, Iowa City, Iowa, United States of America; 4 Veterans Affairs Medical Center, Iowa City, Iowa, United States of America; Indiana University School of Medicine, UNITED STATES

## Abstract

The search for new approaches to treatment and prevention of heart failure is a major challenge in medicine. The adenosine triphosphate-sensitive potassium (K_ATP_) channel has been long associated with the ability to preserve myocardial function and viability under stress. High surface expression of membrane K_ATP_ channels ensures a rapid energy-sparing reduction in action potential duration (APD) in response to metabolic challenges, while cellular signaling that reduces surface K_ATP_ channel expression blunts APD shortening, thus sacrificing energetic efficiency in exchange for greater cellular calcium entry and increased contractile force. In healthy hearts, calcium/calmodulin-dependent protein kinase II (CaMKII) phosphorylates the Kir6.2 K_ATP_ channel subunit initiating a cascade responsible for K_ATP_ channel endocytosis. Here, activation of CaMKII in a transaortic banding (TAB) model of heart failure is coupled with a 35–40% reduction in surface expression of K_ATP_ channels compared to hearts from sham-operated mice. Linkage between K_ATP_ channel expression and CaMKII is verified in isolated cardiomyocytes in which activation of CaMKII results in downregulation of K_ATP_ channel current. Accordingly, shortening of monophasic APD is slowed in response to hypoxia or heart rate acceleration in failing compared to non-failing hearts, a phenomenon previously shown to result in significant increases in oxygen consumption. Even in the absence of coronary artery disease, failing myocardium can be further injured by ischemia due to a mismatch between metabolic supply and demand. Ischemia-reperfusion injury, following ischemic preconditioning, is diminished in hearts with CaMKII inhibition compared to wild-type hearts and this advantage is largely eliminated when myocardial K_ATP_ channel expression is absent, supporting that the myocardial protective benefit of CaMKII inhibition in heart failure may be substantially mediated by K_ATP_ channels. Recognition of CaMKII-dependent downregulation of K_ATP_ channel expression as a mechanism for vulnerability to injury in failing hearts points to strategies targeting this interaction for potential preventives or treatments.

## Introduction

Over the past two decades, there has been considerable progress in the treatment of chronic heart failure yet, even with the best of modern therapy, heart failure is still associated with 5-year mortality rate of 45%-60% [1]. Therefore, the search for new approaches to treatment and prevention of heart failure is one of the major challenges in medicine. One possible target is the adenosine triphosphate-sensitive potassium (K_ATP_) channel that has been long associated with the ability to preserve myocardial function and viability under various stressors [2–7]. The K_ATP_ channel is one of the most abundant cardiac membrane protein complexes and has the unique ability to adjust membrane excitability in response to changes in the energetic status of the cell [4, 5, 8–14]. More recently, K_ATP_ channels have also been shown to be critical regulators of cardiac membrane excitability in response to heart rate acceleration [15]. When activated by increased cellular metabolic demand, K_ATP_ channel-dependent cellular potassium efflux shortens cardiac action potential duration (APD) [2, 3, 8, 9, 16–20]. The outward K_ATP_ current also limits sodium and calcium entry into the cell and thus reduces energy requirements for ion homeostasis and contraction, as well as prolongs the diastolic interval that supports myocardial relaxation and replenishment of resources [2–6, 21–27]. In addition to effects of channel gating, the efficiency of K_ATP_ channel-dependent membrane electrical responses to changes in myocardial energetics has been shown to be highly dependent on the regulation of K_ATP_ channel membrane expression [17, 28–30]. Specifically, a high surface expression of membrane K_ATP_ channels ensures a rapid reduction in APD in response to metabolic challenges thereby providing optimal myocardial energetics, while cellular signaling that reduces surface K_ATP_ channel expression blunts APD shortening, thus sacrificing energetic efficiency in exchange for greater cellular calcium entry and increased contractile force [16, 17, 29–32].

Membrane K_ATP_ channel expression in healthy hearts can be regulated by calcium/calmodulin dependent protein kinase II (CaMKII) [28, 29]. This densely expressed multifunctional kinase targets numerous proteins involved in excitation contraction coupling and excitability to support enhanced cardiac mechanical performance, while its persistent activation under pathophysiological conditions, such as heart failure, promotes cardiomyocyte death and dysfunction [33–36]. In healthy hearts, CaMKII phosphorylates the Kir6.2 pore-forming K_ATP_ channel subunit that initiates a signaling cascade responsible for endocytosis of K_ATP_ channels [29]. This signaling results in a rapid reduction in K_ATP_ channel current capacity that quickly rebounds when CaMKII activation subsides [29]. Excessive and persistent activation of CaMKII, presumably triggered to bolster waning mechanical performance, is a common feature of various types of heart failure [33]. We hypothesize that myocardial vulnerability to injury in failing hearts may be mediated in part by a chronic suppressive effect of CaMKII activation on membrane K_ATP_ channel expression.

Here, we confirm that CaMKII activation is upregulated in a murine model of non-ischemic heart failure induced by transverse aortic banding and that this is associated with a significant reduction in the membrane surface expression of K_ATP_ channels, in their current capacity, and consequently in the responsiveness of ventricular APD shortening under the metabolic stresses of heart rate acceleration and hypoxia. Such changes could aggravate depletion of cardiac energy resources thus contributing to myocardial injury, cell death and heart failure progression, and are consistent with the known beneficial effects on cardiac stress resistance that occur with CaMKII inhibition [33–35, 37–39]. Understanding the interaction between CaMKII activation and cardiac K_ATP_ channel cell membrane expression could present alternative strategies to avoid or treat the excessive myocardial vulnerability to stress that characterizes and promotes heart failure.

## Materials and Methods

All animal protocols conform to the Guide for the Care and Use of Laboratory Animals and were approved by the University of Iowa Institutional Animal Care and Use Committee.

### Heart failure model

Male and female mice aged 10–12 wks were subjected to cardiac pressure overload by transverse aortic banding (TAB) as described [40]. Briefly, mice were anesthetized with ketamine/xylazine (40/5 mg/kg, respectively) by intraperitoneal injection. The mice were intubated with a 16 gauge tube, and ventilated with a small rodent ventilator (Harvard Apparatus, Holliston, MA). A thoracotomy was created between the second and third intercostal space, and the aortic arch visualized. TAB was performed by placing a suture around the aorta and the shank of an 18-gauge needle. The needle was then removed. In sham operated animals, the aortic arch was visualized but not banded. The chest wall was closed, and the pneumothorax evacuated.

### Mouse genetic models

Transgenic mice expressing a specific peptide inhibitor of CaMKII (AC3-I) under control of the cardiac specific *Myh6* promoter [37] and homozygous Kir6.2-KO mice generated by targeted disruption of the *kcnj11* gene [41] were compared to WT littermates. Breeding of AC3-I and Kir6.2-KO mice was performed resulting in offspring homozygous for Kir6.2-KO while expressing the AC3-I transgene (AC3-I/Kir6.2-KO).

### Echocardiography of cardiac function

Two-dimensional transthoracic echocardiography was performed by the University of Iowa Cardiology Animal Phenotyping Core Laboratory using a 30 MHz linear array transducer (Vevo 2100, VisualSonics, Toronto, Canada). Midazolam (.1 mg SC) was used for conscious sedation. Parasternal long- and short-axis views were obtained to evaluate left ventricular function, using the bi-plane area-length method.

### Cardiomyocyte isolation

Single ventricular cardiomyocytes were isolated as described previously [15]. Briefly, hearts were cannulated, then rapidly excised and retrogradely perfused with normal Tyrode solution for 5 min, 3 min with a “low calcium” medium (in mM): 100 NaCl, 10KCl, 1.2 KH_2_PO_4_, 5 MgSO_4_, 20 glucose, 50 taurine, 10 HEPES supplemented with 0.13 CaCl_2_, 2.1 EGTA, then 13 min with low calcium medium supplemented with 1% bovine serum albumin, 0.2 mM CaCl_2_, collagenase (type IV, 22 units/ml, Worthington) and Protease (0.1 mg/ml, Type 16, Sigma). Left ventricles were dissected away and were cut into pieces (~3 × 3 mm). Subsequently the tissue was gently triturated with a glass pipette until dissociated cardiomyocytes were obtained.

### Electrophysiology

Studies were performed using an Axopatch 200B amplifier (Molecular Devices, Sunnyvale, CA) integrated with a Nikon TE2000-U microscope. Experiments were performed at 36–37°C using a temperature controller TC2r (Cell MicroControls, Norfolk, VA). For whole-cell recording, borosilicate glass pipettes (2–3 MΩ) were filled with internal solution (in mM): KCl 140, MgCl_2_ 1, EGTA 5, ATP 5, HEPES-KOH 5, pH 7.3 with KOH. Cardiomyocytes were superfused with Tyrode solution (in mM): NaCl 136.5, KCl 5.4, CaCl_2_ 1, MgCl_2_ 0.53, glucose 5.5, HEPES-NaOH 5, pH 7.4. Whole-cell current traces were obtained in response to 1 s rectangular pulses from a holding potential of −50 mV to test potentials from −80 to +50 mV. For quantification, whole cell K_ATP_ channel current was measured as the difference between baseline and pinacidil- and 2,4-dinitrophenol (DNP)-stimulated current recorded at same voltage potentials. For analysis, only whole cell recordings in which beginning and ending capacitance were within 10% were used. Transmembrane action potentials were recorded in the current-clamp configuration at a stimulation frequency of 1 Hz. All recordings were monitored, sampled, and analyzed using pClamp software (Molecular Devices, Sunnyvale, CA).

### Isolated heart studies

Hearts were extracted from anesthetized mice and retrogradely perfused at 90 mmHg with Krebs-Henseleit buffer bubbled with 95% O_2_/5% CO_2_, at 37°C and pH 7.4. The atrioventricular node was mechanically ablated and hearts paced at 150 ms cycle length (Bloom Electrophysiology, Fischer Imaging Corp., Denver, CO) using a platinum bipolar pacing catheter positioned in the right ventricle (NuMed, Hopkinton, NY). For heart rate acceleration tests, the pacing cycle length was abruptly switched to 90 msec. For hypoxia tests, a separate reservoir of Krebs-Henseleit buffer was bubbled with 95%N_2_/5%CO_2_, at 37°C and pH 7.4. A valve followed by a small bubble chamber and an oxygen sensor immediately above the heart was used to quickly switch the perfusing solutions. Oxygen partial tension was measured in the perfusate prior to heart passage (Model 210, Instech Laboratories, Plymouth Meeting, PA). Coronary flow was measured in series with the aortic cannula (T402, Transonic Systems, Ithica, NY). A monophasic action potential (MAP) probe (EP Technologies, Sunnyvale, CA) was maintained at a single stable position on the LV epicardium, and amplified signals (IsoDam, World Precision Instruments, Sarasota, FL) were acquired at 2 kHz. MAP recordings were analyzed (Clampfit, Molecular Devices, Sunnyvale, CA) for duration at 90% repolarization (MAPD_90_). Steady state changes in MAPD_90_ were calculated using the duration of the MAP just prior to the acceleration of pacing rate or initiation of hypoxia as a reference. MAPs were only analyzed from tracings in which pacing capture was maintained without interruption. All calculations were manually reviewed.

### Western blot

Whole cell protein extracts were used for immunoblotting with CaMKII (Pan, Cell Signaling), phospho-CaMKII (Thr-286, Cell Signaling) and oxidized CaMKII (Ox-CaMKII, gifted from the Anderson laboratory), and anti-GAPDH (Santa Cruz Biotechnologies) antibodies.

### Myocardial ischemia-reperfusion injury

The myocardial ischemia-reperfusion injury model was employed as previously described [28]. Briefly, after allowing an isolated, retrogradely perfused heart to stabilize for 20 minutes, ischemia preconditioning (IP) with two 2 min cycles of global ischemia (stop flow) were followed by 5 min of reperfusion. All hearts were then subjected to 20 min of global ischemia and 45 min reperfusion. At the end of ischemia/reperfusion experiments, hearts were removed from the Langendorff perfusion apparatus and immediately frozen at -20°C. The frozen hearts were cut from apex to base into transverse slices of approximately equal thickness (~0.8 mm). The slices were placed into a small cell culture dish and then incubated in 1% triphenyltetrazolium chloride (TTC) in phosphate buffer (Na_2_HPO_4_: 88 mM, NaH_2_PO_4_ 1.8 mM, pH 7.4) at 37°C for 20 min by rocking the dish. The development of the red formazan pigment relies on the presence of lactate dehydrogenase or NADH in living tissues, while failure to stain red indicates a loss of these constituents from necrotic tissue. After staining, the TTC buffer was replaced by 10% formaldehyde. The slices were fixed for the next 4–6 h before the areas of infarct tissue were determined by ImageJ software. The risk area was the sum of total ventricular area. The infarct size was calculated and presented as percentage of risk area.

### Drugs

The following drugs were purchased from Sigma: isoproterenol, pinacidil, triphenyltetrazolium chloride (TTC) and 2,4-dinitrophenol (DNP).

### Statistical analysis

The data are presented as mean ± the standard error of the mean. Statistical significance is evaluated with the Student’s *t*-test. p<0.05 is considered statistically significant.

## Results

### Decreased contractile function, ventricular enlargement, cellular hypertrophy and action potential duration prolongation occur in the transverse aortic banding model of cardiomyopathy

**Fig 1:**
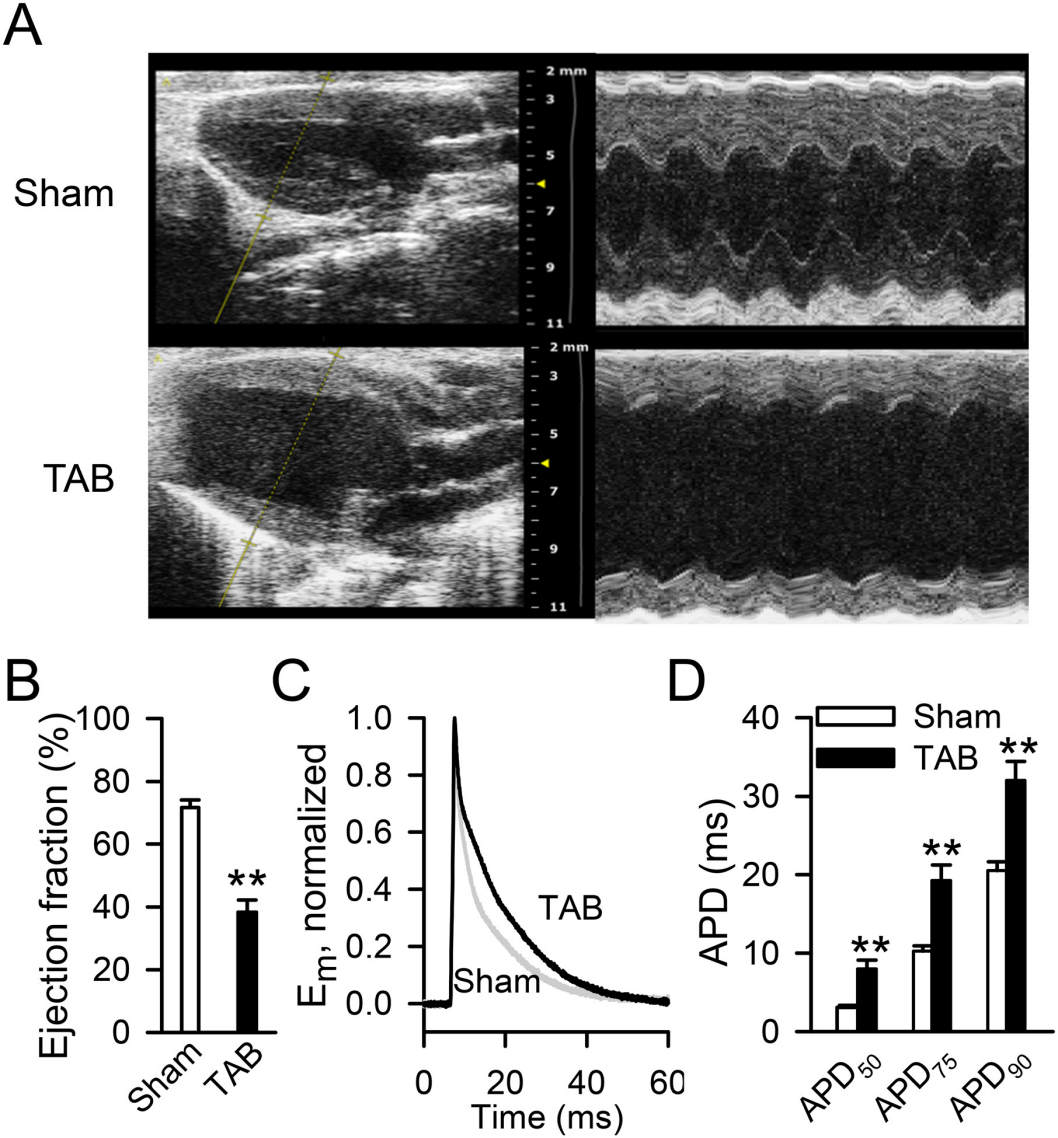
Morphological and electrophysiological changes in transverse aortic banding (TAB) *vs*. sham operated mice. **A)** Representative parasternal long axis echocardiographic images of the ventricles of sham and TAB operated mice. **B)** Summary data of left ventricular ejection fraction (**p<0.01 *vs*. sham). **C)** Representative action potentials recorded in isolated ventricular cardiomyocytes from sham and TAB mice. **D)** Summary data of action potential duration in isolated ventricular myocytes of sham and TAB mice (**p<0.01 *vs*. sham). APD_50_, APD_75_, APD_90_: action potential duration at 50%, 75% and 90% repolarization, respectively. E_m_: membrane potential.

Hearts from mice after transverse aortic banding (TAB) *vs*. sham operation were compared by echocardiogram (Fig 1A). Five to six weeks after operation, the ejection fraction was significantly reduced in the TAB group compared to sham controls (38±4%, n = 12 *vs*. 72±2%, n = 9, p < .01, Fig 1B). Heart weight from TAB operated mice was increased compared to controls (heart weight to body weight ratio of 10.1 mg/g, n = 11 *vs*. 5.9 mg/g, n = 9, p < .01). Isolated ventricular cardiomyocytes from hearts of TAB mice had increased capacitance, representing cell size (291±16, n = 14 *vs*. 158±11 pF, n = 17, p < .01), and longer action potentials than controls (8.0±1.1 *vs*. 3.1±.2, 19.2±2.0 *vs*. 10.3±.6, and 32.0±2.4 *vs*. 20.5±1.1 msec for action potential duration at 50%, 75% and 90% repolarization, respectively, n = 25 cells from 3 TAB mice and 27 cells from 4 sham mice, p < .01 for each comparison, Fig 1C and 1D). These findings indicate that TAB effectively resulted in typical findings of decreased contractile function, ventricular enlargement, cellular hypertrophy and action potential changes associated with heart failure.

### CaMKII is activated in heart failure

**Fig 2:**
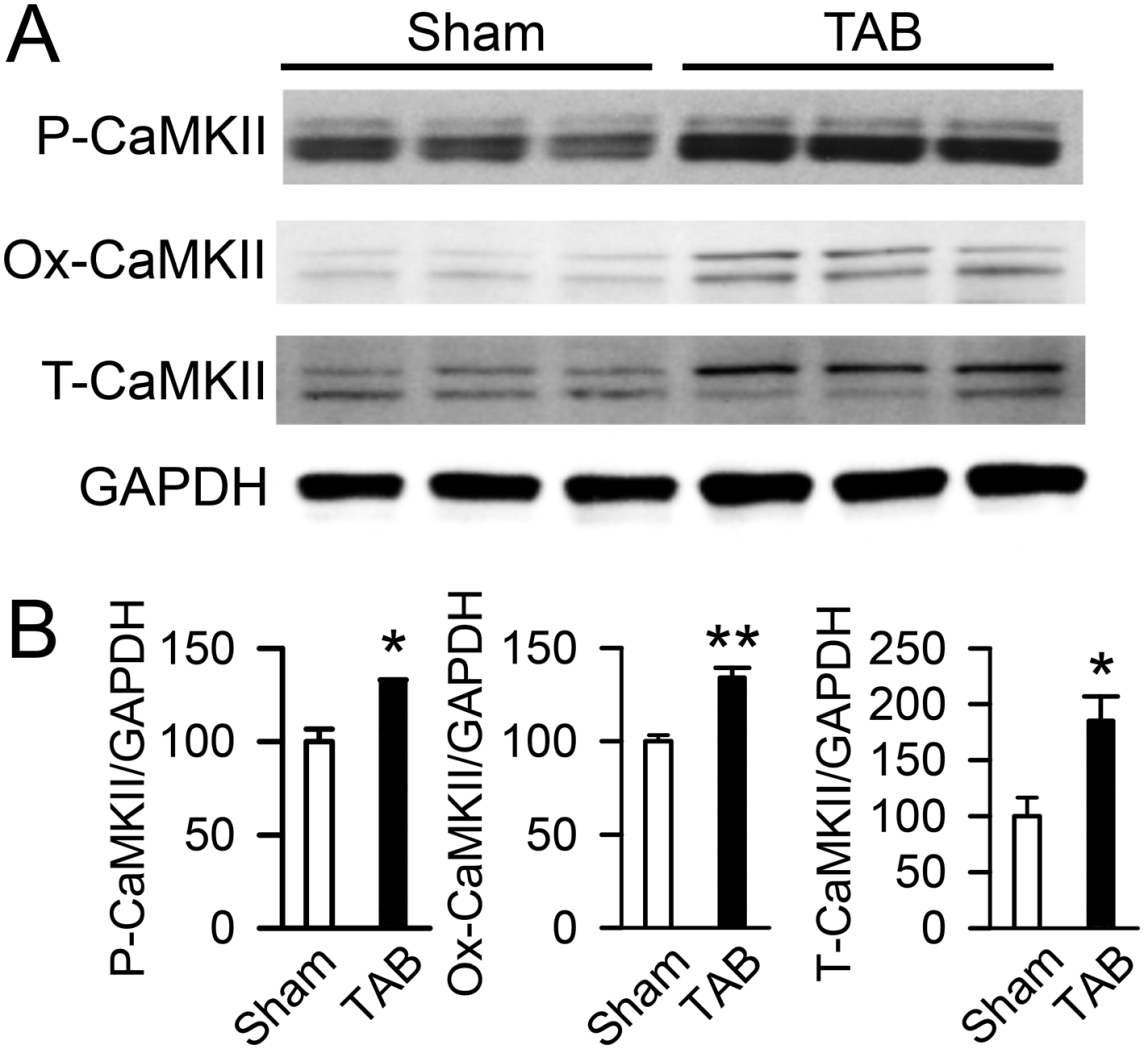
CaMKII is activated in ventricles of hearts following transverse aortic banding. **A)** Representative western blots of phosphorylated CaMKII (P-CaMKII), oxidized CaMKII (Ox-CaMKII), total CaMKII (T-CaMKII) and GAPDH in ventricles of sham and TAB mice as labelled by whole-heart biotinylation. **B)** Summary of P-CaMKII, Ox-CaMKII and T-CaMKII quantification from western blots in ventricles of sham and TAB mice (*p<0.05, **p < .01 *vs*. sham).

It is well established that CaMKII is activated in failing hearts [36, 42–44]. To determine whether the TAB generated model of non-ischemic cardiomyopathy used here recapitulates these reported findings, we assayed CaMKII phosphorylated at residue 286 (P-CaMKII), oxidized CaMKII (Ox-CaMKII), and total CaMKII (T-CaMKII) by western blot in ventricular tissue (Fig 2A). This indicated that expression of P-CaMKII is increased by 32% (132.1±1.0 *vs*. 100.0±6.6 AU, n = 3 each, p < .05, Fig 2B, **left panel**) and Ox-CaMKII is increased by 34% (133.9±5.5 *vs*. 100.0±3.3, n = 3 each, p < .01, Fig 2B, **middle panel**) in hearts of TAB compared to sham mice.

### K_ATP_ channel membrane expression and current density are decreased in failing hearts

**Fig 3:**
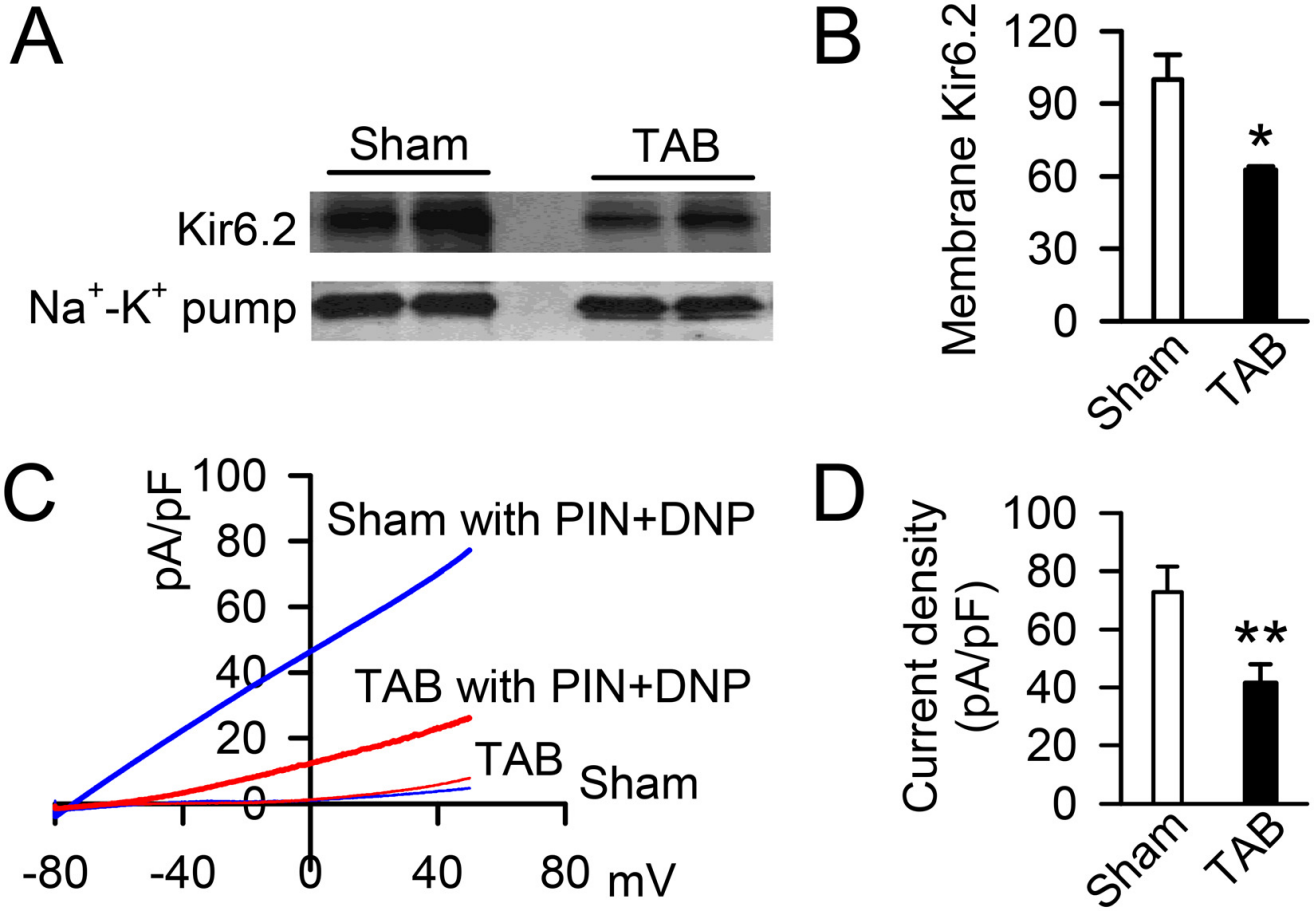
K_ATP_ channel surface expression is down-regulated in ventricles of hearts after transverse aortic banding. A) Representative western blots of the K_ATP_ Kir6.2 subunit and the sodium-potassium pump (Na^+^-K^+^ pump) from the biotinylated membrane fraction of ventricular tissue from isolated hearts of sham and TAB mice. **B)** Summary of ventricular membrane Kir6.2 expression normalized to Na^+^-K^+^ pump expression in hearts of sham and TAB mice (*p<0.05 *vs*. sham). **C)** Representative current profiles of isolated left ventricular myocytes before and after application of the K_ATP_ channel activators, pinacidil (PIN, 100 μM) and 2,4-dinitrophenol (DNP, 200 μM). **D)** Summary of K_ATP_ channel current density (after-before K_ATP_ channel activations) from isolated ventricular cardiomyocytes of sham and TAB mice (**p<0.01 *vs*. sham).

We previously found that activated CaMKII phosphorylates the Kir6.2 subunit and promotes endocytosis of cardiac K_ATP_ channels in healthy hearts [29]. To determine if K_ATP_ channel expression is reduced in association with the persistent CaMKII activation of pressure overload-induced heart failure, we assessed the hearts of TAB and sham operated mice by whole heart biotinylation for the presence of the Kir6.2 subunit on the surface of ventricular cardiomyocytes. We find ventricular surface expression of Kir6.2 (Fig 3A) to be reduced by 37% in hearts from TAB *vs*. sham mice (62.8±1.4 *vs*. 100.0±10.3 AU, n = 3 hearts each, p < .05, Fig 3B). Accordingly, in isolated ventricular myocytes, the K_ATP_ channel current density as stimulated by application of the K_ATP_ channel opener, pinacidil, with the mitochondrial uncoupler, 2,4-dinitrophenol (DNP, Fig 3C) was reduced approximately 40% in TAB compared to sham controls (41.5±6.5 pA/pF, n = 7 cells from 3 TAB mice *vs*. 72.9±8.7 pA/pF, n = 11 cells from 3 sham mice, p < .01, Fig 3D). These data indicate that heart failure and CaMKII activation are associated with a significant reduction in K_ATP_ channel membrane surface expression.

### CaMKII activation results in dynamic reduction of K_ATP_ channel current density in heart failure

**Fig 4:**
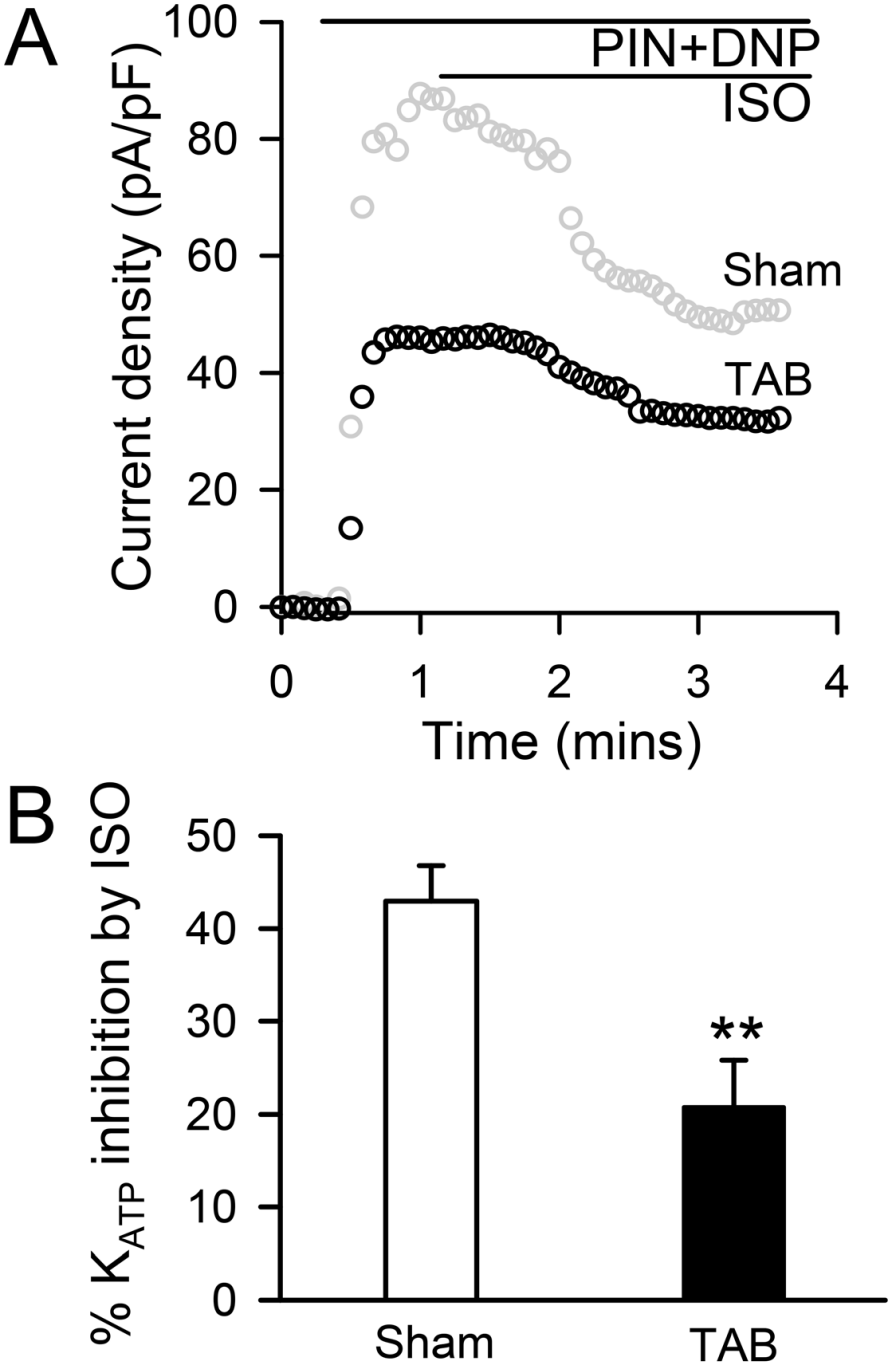
Dynamic downregulation of K_ATP_ channel current density is blunted in ventricular cardiomyocytes of TAB mice. **A)** Representative tracings of pinacidil (PIN, 100 μM)- and 2,4-dinitrophenol (DNP, 200 μM)-stimulated K_ATP_ channel current density in response to isoproterenol (ISO, 1 μM). **B)** Summary of percent inhibition of pinacidil and DNP-stimulated K_ATP_ channel current density by isoproterenol (1 μM) in isolated cardiomyocytes of sham and TAB mice (**p<0.01 *vs*. sham).

We have also previously shown that acute activation of CaMKII in isolated cardiomyocytes by the application of the beta-agonist isoproterenol does not alter K_ATP_ channel gating but results in the endocytosis of K_ATP_ channels causing a rapid decrease in K_ATP_ channel current measured by patch clamp [29]. Here, we tested whether the same dynamic response remains operative in cardiomyocytes from failing TAB hearts. Because K_ATP_ channel current density is already reduced in hearts of TAB *vs*. sham mice, changes in response to isoproterenol are expressed as a % of baseline. We find that the decrease in K_ATP_ channel current density elicited by isoproterenol (Fig 4A) is blunted but still present in ventricular myocytes from hearts of TAB compared to sham control mice (20.8±5.1%, n = 6 cells from 3 TAB mice *vs*. 43.0±3.8%, n = 10 cells from 3 sham mice, p < .01, Fig 4B). Thus these data indicate that the CaMKII-dependent mechanism of K_ATP_ channel membrane expression regulation is pertinent to heart failure conditions.

### Reduction in membrane expression of K_ATP_ channels is associated with a reduced rate of APD shortening

**Fig 5:**
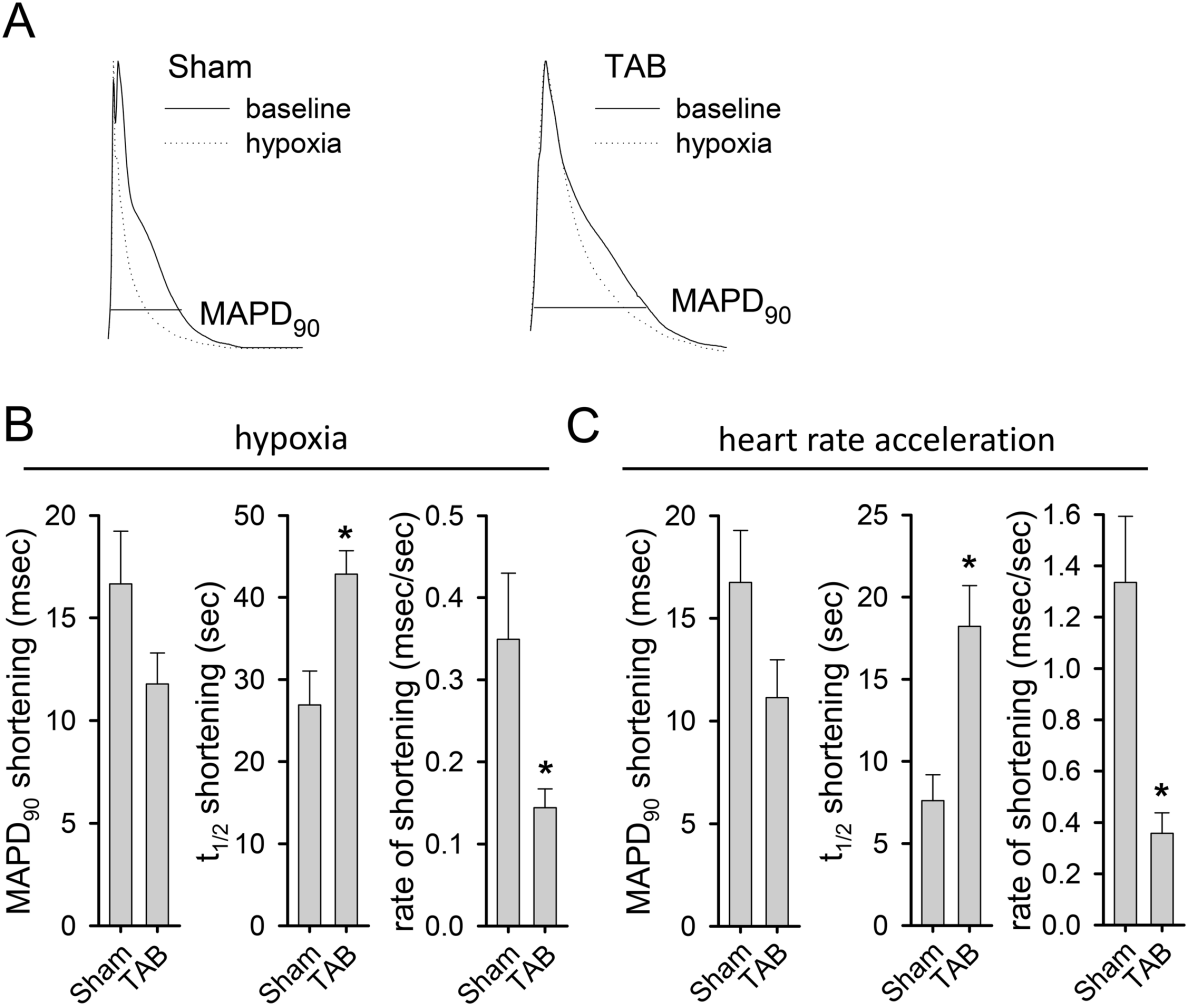
Monophasic action potential duration shortening is blunted in failing hearts. Monophasic action potentials were measured from a single left ventricular epicardial position in isolated hearts from TAB and sham mice before and during exposure to hypoxia or abrupt heart rate acceleration from 400 beats per minute (bpm) to 667 bpm (150 msec cycle length to 90 msec cycle length) driven by pacing. **A)** Representative normalized monophasic action potentials at baseline (solid lines) and in response to hypoxia (dotted lines) from hearts of sham (left panel) and TAB (right panel) mice. A horizontal line designates the point at which monophasic action potential duration at 90% repolarization (MAPD_90_) was measured. **B)** Summary of MAPD_90_ shortening (left), half-time (t_1/2_) of MAPD_90_ shortening (middle), and rate of MAPD_90_ shortening (right) in hearts from sham and TAB mice in response to hypoxia (*p < .05). **C)** Summary of MAPD_90_ shortening (left), half-time (t_1/2_) of MAPD_90_ shortening (middle), and rate of MAPD_90_ shortening (right) in hearts from sham and TAB mice in response to heart rate acceleration (*p < .05).

To assess the physiologic significance of the reduced expression of K_ATP_ channels in failing cardiomyocytes, left ventricular epicardial monophasic action potential duration at 90% repolarization (MAPD_90_) in response to hypoxia (Fig 5A) and heart rate acceleration was measured in isolated hearts from TAB and sham operated mice. Previous data in mice without heart failure indicate that MAPD_90_ shortening and the rate of shortening in response to hypoxia and heart rate acceleration are largely regulated by K_ATP_ channel current [17, 30]. Here, in response to hypoxia, we find that there is a significant slowing in the rate of MAPD_90_ shortening (.14±.02 *vs*. .35±.08 msec/sec, p < .05, Fig 5B **right panel**), also reflected in the greater half-time of maximal shortening (42.9±2.8 vs. 26.9±4.1 sec, p < .05, Fig 5B **middle panel**) for hearts from TAB (n = 10) *vs*. sham controls (n = 9). Similarly, there is a slowing in the rate of MAPD_90_ shortening to abrupt heart rate acceleration (.36±.08 *vs*. 1.34±.26 msec/sec, p < .05, Fig 5C **right panel**), again also reflected in a prolonged half-time of shortening (18.2±2.5 *vs*. 7.6±1.5 sec, p < .05, Fig 5C **middle panel**) in hearts from TAB (n = 12) compared to sham controls (n = 9). In response to both stressors, there is a trend toward slightly less steady state MAPD_90_ shortening that is not statistically significant (11.8±1.5 msec, n = 10 TAB *vs*. 16.7±2.6 msec, n = 9 sham for hypoxia and 11.1±1.8 msec, n = 12 TAB *vs*. 16.8±2.5 msec, n = 9 sham for heart rate acceleration, Fig 5B, **C left panels**). Thus, reduction of membrane K_ATP_ channel expression results in a significant loss of ventricular APD adaptation in failing hearts.

### Disruption of K_ATP_ channel expression eliminates the protective effect of CaMKII inhibition on ischemia-reperfusion injury

**Fig 6:**
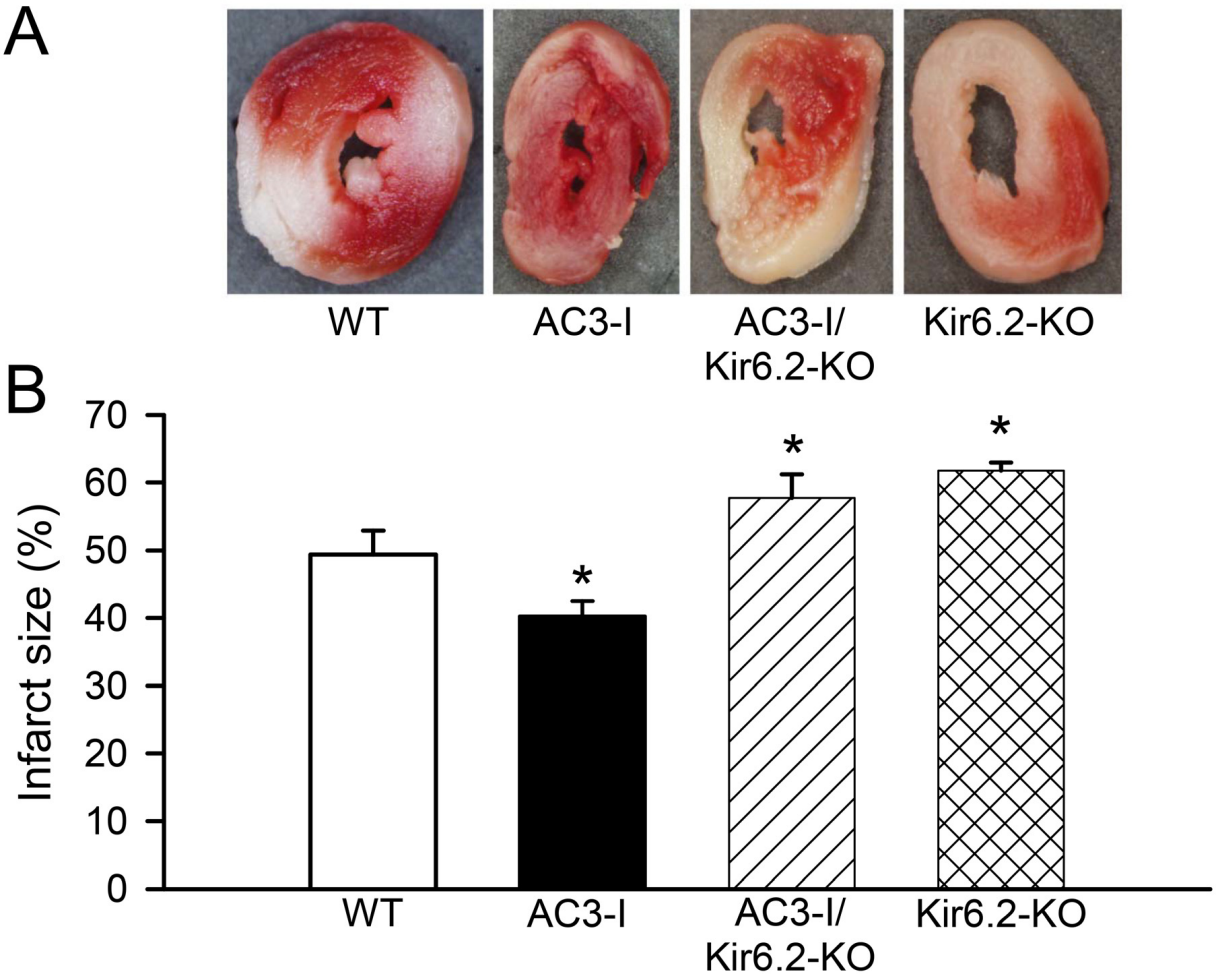
Protective effects of CaMKII inhibition on cardiac ischemia-reperfusion injury is mediated through K_ATP_ channels. **A)** Representative tetrazolium chloride (TTC) staining of fresh ventricular sections after ischemia-reperfusion injury in 4 groups: wild-type (WT), transgenic mice with cardiac-specific expression of a CaMKII inhibitor (AC3-I), mice with knock out of the cardiac K_ATP_ channel pore-forming subunit, Kir6.2 in combined with transgenic cardiac expression of a CaMKII inhibitor (AC3-I/Kir6.2 KO) and mice with knock out of Kir6.2 only (Kir6.2 KO). B) Summary of normalized myocardial infarction size expressed as the average % tissue area stained by TTC in multiple sections of ventricles in response to ischemia-reperfusion (*p < .05 *vs*. WT).

Even in the absence of coronary artery disease, failing myocardium can be injured by ischemia due to a mismatch between metabolic supply and demand [45]. Many studies have demonstrated that CaMKII is activated during cardiac ischemia-reperfusion and contributes significantly to the resulting injury *via* multiple signal cascades [46–56]. Specifically, CaMKII has been shown to be activated by acute ischemia and reperfusion in isolated rodent hearts [49–53, 55, 56]. We have also documented that CaMKII activation triggers the rapid endocytosis of Kir6.2 subunits in the myocardium while CaMKII inhibition results in greater numbers of K_ATP_ channels at the myocyte surface and increased K_ATP_ channel current capacity [29]. This has been confirmed *in situ* with measurement of ventricular myocyte surface Kir6.2 by whole heart biotinylation [29]. Opening of K_ATP_ channels is a well-characterized protective mechanism against cardiac ischemia-reperfusion injury [20, 57, 58]. Previous data indicate that the increased K_ATP_ channel membrane expression in mice transgenically expressing a peptide inhibitor of CaMKII (AC3-I), or increased K_ATP_ channel current induced by K_ATP_ channel openers, correlates with reduced myocardial infarction size following ischemia/reperfusion [28]. Similarly, cardiac CaMKII inhibition or targeted deletion of CaMKIIδ protects against adverse myocardial remodeling following myocardial infarction or TAB [37–39], presumably mediated in part by the loss of CaMKII suppression of K_ATP_ channel expression. Indeed, obtaining comparable degrees of ventricular dysfunction in the presence and absence of CaMKII inhibition in order to replicate such experiments in heart failure would be difficult because CaMKII inhibition significantly affects vulnerability to myocardial dysfunction. Therefore, to assess whether the down-regulation of K_ATP_ channels that we have demonstrated in the hearts of TAB mice may contribute to injury and further heart failure progression, and whether resistance to injury in the presence of CaMKII inhibition can be attributed to increased K_ATP_ channel expression, we assessed ischemia-reperfusion injury in isolated non-failing hearts from WT compared to the AC3-I, Kir6.2-KO and AC3-I/Kir6.2-KO genetic mouse models (Fig 6A). As expected, the area of injury was significantly increased in hearts lacking sarcolemmal K_ATP_ channels (Kir6.2-KO) compared to WT controls (61.7±1.2%, n = 5 *vs*. 49.4±3.5%, n = 8, p < .05, Fig 6B) and significantly reduced in hearts in which a CaMKII inhibitor is constitutively expressed (AC3-I, 40.3±2.2, n = 10, p < .05 *vs*. WT, Fig 6B). Interestingly, the protective benefit of CaMKII inhibition was eliminated in the absence of sarcolemmal K_ATP_ channels (AC3-I/Kir6.2KO, 57.7±3.5%, n = 8, p < .05 *vs*. WT, p = NS *vs*. Kir6.2-KO, Fig 6B), indicating that release of K_ATP_ channel expression from downregulation is a major mechanism for the myocardial protective effects of CaMKII inhibition, at least in response to ischemia-reperfusion, but possibly in response to other pathologic stressors as well.

## Discussion

Here we examine whether chronic, persistent CaMKII activation in failing hearts could promote the heart failure phenotype of action potential prolongation and metabolic vulnerability through an effect on K_ATP_ channel surface expression and current. Indeed, our data indicate a significant reduction of about 35–40% in the myocyte membrane presence of K_ATP_ channels in failing compared to healthy hearts. Since a significant reservoir of sub-membrane K_ATP_ channel subunits is present in cardiomyocytes [29, 31], we use corroborating methods that exclude these spare components from the quantification of membrane surface K_ATP_ channel expression. Specifically, we use the technique of whole heart biotinylation immediately after heart isolation to label only surface-accessible subunits. The result from this method correlates well with the other technique employed—K_ATP_ channel current density measured by whole cell patch clamp in isolated cardiomyocytes.

We have previously demonstrated that the high baseline surface expression of K_ATP_ channels in healthy hearts supports a rapid change in APD in response to myocardial stress that prevents excessive oxygen consumption thereby preserving an optimal myocardial energetic state [2]. Conversely, a significantly slowed adjustment of MAPD_90_ in response to heart rate acceleration or hypoxia occurs with both a decrease in ventricular K_ATP_ channel surface expression of 75–85% in a transgenic mouse model [17, 30] and a 30–50% reduced surface expression in sedentary *vs*. active mice [17], both coupled with proportional increases in oxygen consumption [17]. In the current study, we demonstrate an approximately 30–40% reduction in surface K_ATP_ channels in failing compared to non-failing hearts that is also coupled to a significant slowing in MAPD_90_ shortening with both heart rate acceleration and hypoxia. Given the similar scale to our previous experiments with regard to K_ATP_ channel surface expression deficit and slowing of MAPD_90_ adjustment, a similar effect on oxygen consumption in the TAB mice is expected. Thus we conclude that the reduction in K_ATP_ channel surface expression and sluggish membrane electrical response in the failing hearts of TAB mice is likely sufficient to contribute significantly to the sick metabolic phenotype of heart failure.

We have previously established that K_ATP_ channel expression on the surface of healthy cardiomyocytes is directly affected by CaMKII activation due to phosphorylation of the Kir6.2 subunit by CaMKII that then initiates endocytosis of channel subunits [29]. Yet in heart failure, where remodeling re-orders numerous cell processes, it is possible that the loss of surface K_ATP_ channels at baseline in failing hearts is unrelated to the demonstrated CaMKII activation. However, we find that the diminished K_ATP_ channel current density in the failing hearts can be further downregulated, albeit in a blunted fashion, in response to activation of CaMKII by isoproterenol. We interpret this to indicate that, despite many profound remodeling effects in heart failure, the mechanism of CaMKII-induced internalization of K_ATP_ channels is still operable. Furthermore, the chronic activation of CaMKII in the failing TAB hearts and the consequent low baseline surface expression of K_ATP_ channels probably preclude further vigorous K_ATP_ channel downregulation since there is likely little remaining dynamic range for either CaMKII activation or K_ATP_ channel internalization.

In non-failing hearts, we demonstrate that CaMKII inhibition attenuates ischemia-reperfusion injury—an effect that is eliminated by knockout of Kir6.2—corroborating a previous pharmacologic study [28] and indicating that this cardioprotective CaMKII inhibition-driven phenomenon is mediated substantially by K_ATP_ channels. Since our data support that K_ATP_ channel surface expression remains both chronically and acutely under the influence of CaMKII, it is reasonable to expect that a protective effect of CaMKII inhibition in failing hearts is also largely mediated by a release of K_ATP_ channel expression from suppression. Further studies with induction of CaMKII inhibition after the establishment of heart failure, with and without Kir6.2-KO, may confirm these findings.

Recognition of a CaMKII-dependent downregulation of K_ATP_ channel expression as a mechanism of heart failure development and progression points to strategies targeting this interaction for potential preventives or treatments. Indeed, CaMKII inhibition has been scrutinized as a promising therapeutic approach in heart failure. However, diverse tissue expression and pleiotropic vital functions of CaMKII have made it a problematic target. Meanwhile, the presently available K_ATP_ channel openers do not have cardiac specificity and their therapeutic use is limited due to potent effects on vasculature tone causing hypotension [59–61]. Given these limitations, deciphering the interaction between CaMKII and K_ATP_ channel surface expression could provide for development of more specific approaches to securing myocardial metabolic well-being and preventing or treating heart failure.

We recognize that the alteration in K_ATP_ channel surface expression identified in this study is one element in a complex array of ion channel adaptations identified in heart failure. Indeed, heart failure has been associated with numerous modifications of ion channels, the precise profile of which can be specific to the myocardial layer, stage and etiology of heart failure [62–64]. Furthermore, interactions between ion channels contribute to the electrophysiologic phenotype of various types of heart failure [62, 63]. It is likely that K_ATP_ channel surface expression in heart failure is similarly non-uniform across layers, models and stage and that the impact of their downregulation depends in part on interactions between K_ATP_ channels and other ion channels. Further study will be needed to determine whether our findings here in a TAB model will translate to other etiologies and severities of heart failure.

Whether the demonstrated perturbation in K_ATP_ channel surface expression, and its effect on APD shortening and myocardial energetics, is relevant to human disease also remains to be tested. One might argue that the short cycle length in rodents necessitates strong repolarizing currents to prevent action potential fusion and that rodents would thus be more dependent on K_ATP_ channel opening under stressful conditions than larger mammals. However a comparison of studies across animal models indicates that the cardioprotective effect of K_ATP_ channels occurs within a broad range of ion channel profiles and associated shapes and durations of action potentials [65]. Furthermore, the current study and previous studies [17, 30] identify *changes* in APD related to stress as important for the K_ATP_ channel effect on myocardial energetics and cardioprotection. The question of applicability of our findings to human heart failure is also partially addressed by a previous study indicating a defect in K_ATP_ channel gating as a potential cause of familial cardiomyopathy [27]. While that study did not identify deficient K_ATP_ channel expression, it reinforces the concept that insufficient K_ATP_ channel current, which could be caused by either gating or expression defects, may predispose human myocardium to injury and mechanical failure.

## Conclusions

Our data indicate that ventricular K_ATP_ channel surface expression is under the control of CaMKII in failing hearts and that the chronic CaMKII activation in this disease state suppresses K_ATP_ channel surface expression sufficiently to incur a failure of membrane electrical responsiveness associated with negative metabolic consequences. Our data support that this mechanism underlies an increased risk of myocardial injury that can translate to heart failure progression.

## References

[1] Huffman MD, Berry JD, Ning H, Dyer AR, Garside DB, Cai X, et al Lifetime risk for heart failure among white and black Americans: cardiovascular lifetime risk pooling project. J Am Coll Cardiol. 2013;61(14):1510–7. 10.1016/j.jacc.2013.01.022 23500287 PMC3618527

[2] Zingman LV, Hodgson DM, Bast PH, Kane GC, Perez-Terzic C, Gumina RJ, et al Kir6.2 is required for adaptation to stress. ProcNatlAcadSciUSA. 2002;99(20):13278. 10.1073/pnas.212315199 PMC130624 12271142

[3] Hodgson DM, Zingman LV, Kane GC, Perez-Terzic C, Bienengraeber M, Ozcan C, et al Cellular remodeling in heart failure disrupts K(ATP) channel-dependent stress tolerance. Embo J. 2003;22(8):1732 12682006 10.1093/emboj/cdg192 PMC154482

[4] Flagg TP, Enkvetchakul D, Koster JC, Nichols CG. Muscle KATP channels: recent insights to energy sensing and myoprotection. PhysiolRev. 2010;90(3):799. 10.1152/physrev.00027.2009 PMC3125986 20664073

[5] Seino S, Miki T. Physiological and pathophysiological roles of ATP-sensitive K+ channels. ProgBiophysMolBiol. 2003;81(2):133. 10.1016/s0079-6107(02)00053-6 12565699

[6] Kane GC, Behfar A, Dyer RB, O'Cochlain DF, Liu XK, Hodgson DM, et al KCNJ11 gene knockout of the Kir6.2 KATP channel causes maladaptive remodeling and heart failure in hypertension. HumMolGenet. 2006;15(15):2285. 10.1093/hmg/ddl154 16782803

[7] Yamada S, Kane GC, Behfar A, Liu XK, Dyer RB, Faustino RS, et al Protection conferred by myocardial ATP-sensitive K+ channels in pressure overload-induced congestive heart failure revealed in KCNJ11 Kir6.2-null mutant. JPhysiol. 2006;577(Pt 3):1053. 17038430 10.1113/jphysiol.2006.119511 PMC1890387

[8] Weiss JN, Venkatesh N. Metabolic regulation of cardiac ATP-sensitive K+ channels. CardiovascDrugs Ther. 1993;7 Suppl 3:499. 10.1007/BF00877614 8251419

[9] Alekseev AE, Hodgson DM, Karger AB, Park S, Zingman LV, Terzic A. ATP-sensitive K+ channel channel/enzyme multimer: metabolic gating in the heart. JMolCellCardiol. 2005;38(6):895. 10.1016/j.yjmcc.2005.02.022 PMC2736952 15910874

[10] Noma A. ATP-regulated K+ channels in cardiac muscle. Nature. 1983;305(5930):147 6310409 10.1038/305147a0

[11] Aguilar-Bryan L, Bryan J. Molecular biology of adenosine triphosphate-sensitive potassium channels. EndocrRev. 1999;20(2):101. 10.1210/edrv.20.2.0361 10204114

[12] Ashcroft FM. Adenosine 5'-triphosphate-sensitive potassium channels. AnnuRevNeurosci. 1988;11:97. 10.1146/annurev.ne.11.030188.000525 2452599

[13] Lederer WJ, Nichols CG. Nucleotide modulation of the activity of rat heart ATP-sensitive K+ channels in isolated membrane patches. JPhysiol. 1989;419:193. 2621629 10.1113/jphysiol.1989.sp017869 PMC1190004

[14] Shyng S, Ferrigni T, Nichols CG. Regulation of KATP channel activity by diazoxide and MgADP. Distinct functions of the two nucleotide binding folds of the sulfonylurea receptor. JGenPhysiol. 1997;110(6):643. 10.1085/jgp.110.6.643 PMC2229399 9382893

[15] Zingman LV, Zhu Z, Sierra A, Stepniak E, Burnett CM, Maksymov G, et al Exercise-induced expression of cardiac ATP-sensitive potassium channels promotes action potential shortening and energy conservation. Journal of molecular and cellular cardiology. 2011;51(1):72–81. 10.1016/j.yjmcc.2011.03.010 21439969 PMC3103621

[16] Alekseev AE, Reyes S, Yamada S, Hodgson-Zingman DM, Sattiraju S, Zhu Z, et al Sarcolemmal ATP-sensitive K(+) channels control energy expenditure determining body weight. CellMetab. 2010;11(1):58. 10.1016/j.cmet.2009.11.009 PMC2849280 20074528

[17] Zingman LV, Zhu Z, Sierra A, Stepniak E, Burnett CM, Maksymov G, et al Exercise-induced expression of cardiac ATP-sensitive potassium channels promotes action potential shortening and energy conservation. JMolCellCardiol. 2011;51(1):72. 10.1016/j.yjmcc.2011.03.010 PMC3103621 21439969

[18] Tong X, Porter LM, Liu G, Dhar-Chowdhury P, Srivastava S, Pountney DJ, et al Consequences of cardiac myocyte-specific ablation of KATP channels in transgenic mice expressing dominant negative Kir6 subunits. AmJPhysiolHeart CircPhysiol. 2006;291(2):H543. 10.1152/ajpheart.00051.2006 PMC2950019 16501027

[19] Nichols CG, Ripoll C, Lederer WJ. ATP-sensitive potassium channel modulation of the guinea pig ventricular action potential and contraction. CircRes. 1991;68(1):280. 10.1161/01.res.68.1.280 1984868

[20] Zingman LV, Alekseev AE, Hodgson-Zingman DM, Terzic A. ATP-sensitive potassium channels: metabolic sensing and cardioprotection. JApplPhysiol. 2007;103(5):1888. 10.1152/japplphysiol.00747.2007 17641217

[21] Balaban RS. Cardiac energy metabolism homeostasis: role of cytosolic calcium. JMolCellCardiol. 2002;34(10):1259. 10.1006/jmcc.2002.2082 12392982

[22] Moore RL. Myocardial KATP channels are critical to Ca2+ homeostasis in the metabolically stressed heart in vivo. AmJPhysiolHeart CircPhysiol. 2007;292(4):H1692. 10.1152/ajpheart.00076.2007 17259443

[23] Eisner DA, Dibb KM, Trafford AW. The mechanism and significance of the slow changes of ventricular action potential duration following a change of heart rate. ExpPhysiol. 2009;94(5):520. 10.1113/expphysiol.2008.044008 19270038

[24] Carmeliet E. Intracellular Ca(2+) concentration and rate adaptation of the cardiac action potential. Cell Calcium. 2004;35(6):557 15110146 10.1016/j.ceca.2004.01.010

[25] Liu XK, Yamada S, Kane GC, Alekseev AE, Hodgson DM, O'Cochlain F, et al Genetic disruption of Kir6.2, the pore-forming subunit of ATP-sensitive K+ channel, predisposes to catecholamine-induced ventricular dysrhythmia. Diabetes. 2004;53 Suppl 3:S165 15561906 10.2337/diabetes.53.suppl_3.s165

[26] Gumina RJ, O'Cochlain DF, Kurtz CE, Bast P, Pucar D, Mishra P, et al KATP channel knockout worsens myocardial calcium stress load in vivo and impairs recovery in stunned heart. AmJPhysiolHeart CircPhysiol. 2007;292(4):H1706. 10.1152/ajpheart.01305.2006 17189350

[27] Bienengraeber M, Olson TM, Selivanov VA, Kathmann EC, O'Cochlain F, Gao F, et al ABCC9 mutations identified in human dilated cardiomyopathy disrupt catalytic KATP channel gating. NatGenet. 2004;36(4):382. 10.1038/ng1329 PMC1995438 15034580

[28] Li J, Marionneau C, Koval O, Zingman L, Mohler PJ, Nerbonne JM, et al Calmodulin kinase II inhibition enhances ischemic preconditioning by augmenting ATP-sensitive K+ current. Channels (Austin). 2007;1(5):387. 18690039 10.4161/chan.5449

[29] Sierra A, Zhu Z, Sapay N, Sharotri V, Kline CF, Luczak ED, et al Regulation of cardiac ATP-sensitive potassium channel surface expression by calcium/calmodulin-dependent protein kinase II. JBiolChem. 2013;288(3):1568. 10.1074/jbc.M112.429548 PMC3548467 23223335

[30] Zhu Z, Burnett CM, Maksymov G, Stepniak E, Sierra A, Subbotina E, et al Reduction in number of sarcolemmal KATP channels slows cardiac action potential duration shortening under hypoxia. BiochemBiophysResCommun. 2011;415(4):637. 10.1016/j.bbrc.2011.10.125 PMC3230708 22079630

[31] Bao L, Hadjiolova K, Coetzee WA, Rindler MJ. Endosomal KATP channels as a reservoir after myocardial ischemia: a role for SUR2 subunits. AmJPhysiolHeart CircPhysiol. 2011;300(1):H262. 10.1152/ajpheart.00857.2010 PMC3023244 20971764

[32] Zhu Z, Sierra A, Burnett CM, Chen B, Subbotina E, Koganti SR, et al Sarcolemmal ATP-sensitive potassium channels modulate skeletal muscle function under low-intensity workloads. J Gen Physiol. 2014;143(1):119–34. 10.1085/jgp.201311063 24344248 PMC3874572

[33] Anderson ME, Brown JH, Bers DM. CaMKII in myocardial hypertrophy and heart failure. JMolCellCardiol. 2011. 10.1016/j.yjmcc.2011.01.012 PMC3158288 21276796

[34] Neef S, Maier LS. Remodeling of excitation-contraction coupling in the heart: inhibition of sarcoplasmic reticulum Ca(2+) leak as a novel therapeutic approach. CurrHeart FailRep. 2007;4(1):11. 10.1007/s11897-007-0020-7 17386180

[35] Maier LS. Role of CaMKII for signaling and regulation in the heart. FrontBiosci. 2009;14:486. 10.2741/3257 19273080

[36] Kushnir A, Shan J, Betzenhauser MJ, Reiken S, Marks AR. Role of CaMKIIdelta phosphorylation of the cardiac ryanodine receptor in the force frequency relationship and heart failure. ProcNatlAcadSciUSA. 2010;107(22):10274. 10.1073/pnas.1005843107 PMC2890457 20479242

[37] Zhang R, Khoo MS, Wu Y, Yang Y, Grueter CE, Ni G, et al Calmodulin kinase II inhibition protects against structural heart disease. NatMed. 2005;11(4):409. 10.1038/nm1215 15793582

[38] Backs J, Backs T, Neef S, Kreusser MM, Lehmann LH, Patrick DM, et al The delta isoform of CaM kinase II is required for pathological cardiac hypertrophy and remodeling after pressure overload. ProcNatlAcadSciUSA. 2009;106(7):2342. 10.1073/pnas.0813013106 PMC2650158 19179290

[39] Ling H, Zhang T, Pereira L, Means CK, Cheng H, Gu Y, et al Requirement for Ca2+/calmodulin-dependent kinase II in the transition from pressure overload-induced cardiac hypertrophy to heart failure in mice. JClinInvest. 2009;119(5):1230. 10.1172/JCI38022 PMC2673879 19381018

[40] Wei S, Guo A, Chen B, Kutschke W, Xie YP, Zimmerman K, et al T-tubule remodeling during transition from hypertrophy to heart failure. Circulation research. 2010;107(4):520–31. 10.1161/CIRCRESAHA.109.212324 20576937 PMC2927862

[41] Miki T, Nagashima K, Tashiro F, Kotake K, Yoshitomi H, Tamamoto A, et al Defective insulin secretion and enhanced insulin action in KATP channel-deficient mice. ProcNatlAcadSciUSA. 1998;95(18):10402. 10.1073/pnas.95.18.10402 PMC27906 9724715

[42] Luo M, Guan X, Luczak ED, Lang D, Kutschke W, Gao Z, et al Diabetes increases mortality after myocardial infarction by oxidizing CaMKII. J Clin Invest. 2013;123(3):1262–74. 10.1172/JCI65268 23426181 PMC3673230

[43] Purohit A, Rokita AG, Guan X, Chen B, Koval OM, Voigt N, et al Oxidized Ca(2+)/calmodulin-dependent protein kinase II triggers atrial fibrillation. Circulation. 2013;128(16):1748–57. 10.1161/CIRCULATIONAHA.113.003313 24030498 PMC3876034

[44] Sossalla S, Fluschnik N, Schotola H, Ort KR, Neef S, Schulte T, et al Inhibition of elevated Ca2+/calmodulin-dependent protein kinase II improves contractility in human failing myocardium. Circ Res. 2010;107(9):1150–61. 10.1161/CIRCRESAHA.110.220418 20814023

[45] Kronenberg MW, Cohen GI, Leonen MF, Mladsi TA, Di Carli MF. Myocardial oxidative metabolic supply-demand relationships in patients with nonischemic dilated cardiomyopathy. J Nucl Cardiol. 2006;13(4):544–53. 16919578 10.1016/j.nuclcard.2006.04.002

[46] Di Carlo MN, Said M, Ling H, Valverde CA, De Giusti VC, Sommese L, et al CaMKII-dependent phosphorylation of cardiac ryanodine receptors regulates cell death in cardiac ischemia/reperfusion injury. J Mol Cell Cardiol. 2014;74:274–83. 10.1016/j.yjmcc.2014.06.004 24949568 PMC4131282

[47] Ling H, Gray CB, Zambon AC, Grimm M, Gu Y, Dalton N, et al Ca2+/Calmodulin-dependent protein kinase II delta mediates myocardial ischemia/reperfusion injury through nuclear factor-kappaB. Circ Res. 2013;112(6):935–44. 10.1161/CIRCRESAHA.112.276915 23388157 PMC3673710

[48] Bell JR, Vila-Petroff M, Delbridge LM. CaMKII-dependent responses to ischemia and reperfusion challenges in the heart. Frontiers in pharmacology. 2014;5:96 10.3389/fphar.2014.00096 24834054 PMC4018566

[49] Said M, Becerra R, Valverde CA, Kaetzel MA, Dedman JR, Mundina-Weilenmann C, et al Calcium-calmodulin dependent protein kinase II (CaMKII): a main signal responsible for early reperfusion arrhythmias. J Mol Cell Cardiol. 2011;51(6):936–44. 10.1016/j.yjmcc.2011.08.010 21888910 PMC3208750

[50] Said M, Vittone L, Mundina-Weilenmann C, Ferrero P, Kranias EG, Mattiazzi A. Role of dual-site phospholamban phosphorylation in the stunned heart: insights from phospholamban site-specific mutants. American journal of physiology Heart and circulatory physiology. 2003;285(3):H1198–205. 12763747 10.1152/ajpheart.00209.2003

[51] Vittone L, Mundina-Weilenmann C, Said M, Ferrero P, Mattiazzi A. Time course and mechanisms of phosphorylation of phospholamban residues in ischemia-reperfused rat hearts. Dissociation of phospholamban phosphorylation pathways. J Mol Cell Cardiol. 2002;34(1):39–50. 11812163 10.1006/jmcc.2001.1488

[52] Salas MA, Valverde CA, Sanchez G, Said M, Rodriguez JS, Portiansky EL, et al The signalling pathway of CaMKII-mediated apoptosis and necrosis in the ischemia/reperfusion injury. J Mol Cell Cardiol. 2010;48(6):1298–306. 10.1016/j.yjmcc.2009.12.015 20060004 PMC2866824

[53] Valverde CA, Mundina-Weilenmann C, Reyes M, Kranias EG, Escobar AL, Mattiazzi A. Phospholamban phosphorylation sites enhance the recovery of intracellular Ca2+ after perfusion arrest in isolated, perfused mouse heart. Cardiovasc Res. 2006;70(2):335–45. 16516179 10.1016/j.cardiores.2006.01.018

[54] Vila-Petroff M, Mundina-Weilenmann C, Lezcano N, Snabaitis AK, Huergo MA, Valverde CA, et al Ca(2+)/calmodulin-dependent protein kinase II contributes to intracellular pH recovery from acidosis via Na(+)/H(+) exchanger activation. J Mol Cell Cardiol. 2010;49(1):106–12. 10.1016/j.yjmcc.2009.12.007 20026127 PMC2883686

[55] Hidalgo CG, Chung CS, Saripalli C, Methawasin M, Hutchinson KR, Tsaprailis G, et al The multifunctional Ca(2+)/calmodulin-dependent protein kinase II delta (CaMKIIdelta) phosphorylates cardiac titin's spring elements. J Mol Cell Cardiol. 2013;54:90–7. 10.1016/j.yjmcc.2012.11.012 23220127 PMC3535572

[56] Vila-Petroff M, Salas MA, Said M, Valverde CA, Sapia L, Portiansky E, et al CaMKII inhibition protects against necrosis and apoptosis in irreversible ischemia-reperfusion injury. Cardiovasc Res. 2007;73(4):689–98. 17217936 10.1016/j.cardiores.2006.12.003

[57] Elrod JW, Harrell M, Flagg TP, Gundewar S, Magnuson MA, Nichols CG, et al Role of sulfonylurea receptor type 1 subunits of ATP-sensitive potassium channels in myocardial ischemia/reperfusion injury. Circulation. 2008;117(11):1405–13. 10.1161/CIRCULATIONAHA.107.745539 18316485

[58] Suzuki M, Sasaki N, Miki T, Sakamoto N, Ohmoto-Sekine Y, Tamagawa M, et al Role of sarcolemmal K(ATP) channels in cardioprotection against ischemia/reperfusion injury in mice. JClinInvest. 2002;109(4):509. 10.1172/JCI14270 PMC150878 11854323

[59] Moreau C, Jacquet H, Prost AL, D'Hahan N, Vivaudou M. The molecular basis of the specificity of action of K(ATP) channel openers. Embo J. 2000;19(24):6644 11118199 10.1093/emboj/19.24.6644 PMC305901

[60] Ashcroft FM, Gribble FM. New windows on the mechanism of action of K(ATP) channel openers. Trends PharmacolSci. 2000;21(11):439. 10.1016/s0165-6147(00)01563-7 11121575

[61] Jahangir A, Terzic A. K(ATP) channel therapeutics at the bedside. JMolCellCardiol. 2005;39(1):99. 10.1016/j.yjmcc.2005.04.006 PMC2743392 15953614

[62] Coronel R, Wilders R, Verkerk AO, Wiegerinck RF, Benoist D, Bernus O. Electrophysiological changes in heart failure and their implications for arrhythmogenesis. Biochim Biophys Acta. 2013;1832(12):2432–41. 10.1016/j.bbadis.2013.04.002 23579069

[63] Wang Y, Hill JA. Electrophysiological remodeling in heart failure. J Mol Cell Cardiol. 2010;48(4):619–32. 10.1016/j.yjmcc.2010.01.009 20096285 PMC2879059

[64] Wickenden AD, Kaprielian R, Kassiri Z, Tsoporis JN, Tsushima R, Fishman GI, et al The role of action potential prolongation and altered intracellular calcium handling in the pathogenesis of heart failure. Cardiovasc Res. 1998;37(2):312–23. 9614488 10.1016/s0008-6363(97)00256-3

[65] Foster MN, Coetzee WA. KATP Channels in the Cardiovascular System. Physiol Rev. 2016;96(1):177–252. 10.1152/physrev.00003.2015 26660852 PMC4698399

